# Assessment of patients’ perception of the use of an implantable cardiac electronic device

**DOI:** 10.1590/0034-7167-2025-0299

**Published:** 2026-09-18

**Authors:** Isabela Santana Oliveira, Renata Elizabete Pagotti da Fonseca, Joyce Aparecida Turco Machado Francisco, Suellen Rodrigues de Oliveira Maier, André Filipe Morais Pinto Novo, Carina Aparecida Marosti Dessotte

**Affiliations:** I Universidade de São Paulo. Ribeirão Preto, São Paulo, Brazil; II Universidade Federal de São Carlos. São Carlos, São Paulo, Brazil; III Universidade Federal de Rondonópolis. Rondonópolis, Mato Grosso, Brazil; IV Instituto Politécnico de Bragança. Bragança, Portugal

**Keywords:** Pacemaker, Perception, Perioperative Nursing, Arrhythmias, Cardiac, Cardiovascular Diseases., Marcapaso Artificial, Percepción, Enfermería Perioperatoria, Arritmias Cardíacas, Enfermedades Cardiovasculares.

## Abstract

**Objectives:**

to assess patients’ perceptions after pacemaker implantation.

**Methods:**

a descriptive, cross-sectional observational study was conducted at a university hospital in the countryside of São Paulo, with a consecutive and non-probabilistic sample of adult patients with pacemakers. A questionnaire was used to assess patients’ perceptions of pacemakers, with data analysis obtained by simple frequency and percentage of responses.

**Results:**

of the 76 patients interviewed, the majority were female, had a partner, were inactive, had low levels of education and family income, with an average age of 68.3 years. The main reason for implant recommendation was complete atrioventricular block. Most (73.7%) reported no feelings of physical impairment, nor did they feel depressed or anxious. They also reported no changes in body image, daily activities, or leisure activities. Furthermore, they demonstrated a high level of confidence regarding the device use (61.8%).

**Conclusions:**

patients positively assessed the use of pacemakers.

## RESUMEN

**Objetivos:**

evaluar las percepciones de los pacientes tras la implantación del marcapasos.

**Métodos:**

estudio observacional descriptivo y transversal en hospital universitario del interior de São Paulo, con una muestra consecutiva y no probabilística de pacientes adultos con marcapasos. Se utilizó un cuestionario para evaluar las percepciones de los pacientes sobre el marcapasos, y el análisis de datos se realizó mediante frecuencia simple y porcentaje de respuestas.

**Resultados:**

de los 76 pacientes entrevistados, la mayoría eran mujeres, tenían pareja, eran inactivas, presentaban bajos niveles de educación e ingresos familiares, con una edad promedio de 68,3 años. La principal razón para la indicación del implante fue el bloqueo auriculoventricular completo. La mayoría (73,7%) no reportó ninguna sensación de discapacidad física, ni tampoco se sintió deprimida o ansiosa. Tampoco reportaron cambios en su imagen corporal, actividades diarias o de ocio. Además, demostraron un alto nivel de confianza en el uso del dispositivo (61,8%).

**Conclusiones:**

los pacientes valoraron positivamente el uso del marcapasos.

## INTRODUCTION

Cardiovascular diseases (CVDs) are the leading cause of death worldwide. Between 1990 and 2022, deaths from CVDs increased from 12.4 million to 19.8 million, while the global population grew by 51%^(1)^. In Brazil, CVDs also rank first among causes of death. After losing their position to COVID-19 in 2021, heart and circulatory system diseases returned to the top, with approximately 400,000 Brazilians dying in 2022 due to 18 types of CVDs, according to the report “Global burden of disease and cardiovascular risk factors”^(2)^.

Currently in Brazil, it is estimated that more than 20 million people have some type of cardiac arrhythmia^(3)^. According to data from the Brazilian Registry of Pacemakers, Resynchronizers and Defibrillators (*Registro Brasileiro de Marcapassos, Ressincronizadores e Desfibriladores*), between July 12, 2024, and August 12, 2024, 1,723 pacemaker (PM) implants were performed nationwide^(4)^. From January to June 2024, a total of 435,211 PM implants were performed, with the Southeast region performing the highest number (196,377), followed by the South (101,713), Northeast (82,035), Midwest (40,203), and North (14,883)^(5)^.

Patients indicated for PM implantation present severe CVD incompatible with life, and implantation is often one of the last therapeutic options. After implantation, complications may arise both from cardiac deterioration and from the device itself. In addition to infections, there are risks of induced cardiomyopathy, tricuspid regurgitation, cardiac perforation, hemothorax, and pneumothorax^(6)^. These problems can be minimized by adherence to specific care to preserve the device^(7)^.

Global estimates indicate that 1.25 million permanent PMs are implanted annually, and this number is expected to increase in the coming years^(8)^. Given this global trend, special attention should be paid to the impact of PM therapy on patients’ quality of life and their perception of this therapy.

Patients with older PMs may experience environmental interference which, although rare, may affect their functioning. These interferences may cause inhibition of stimulation, inappropriate triggering, asynchronous function, programming changes, and inappropriate acceleration. Depending on intensity, they may result in transient dysfunction, irreversible generator damage, arrhythmias, and changes at the electrode-heart junction^(9)^.

Although such interferences may occur, the PM itself does not impose restrictions on physical activity, allowing patients to return to daily life. Lack of knowledge or misunderstanding may lead to unnecessary restrictions or secondary risks related to interference, impairing patients’ quality of life^(10)^.

From a social perspective, maintaining quality of life creates stability and equality among members of society. For patients, quality of life reflects what actually occurs in their daily lives and indicates the gap between expectations and reality. From a clinical perspective, quality of life provides essential information for healthcare professionals when planning patient-centered care^(11)^. Therefore, it is important to examine patients’ perceptions regarding PM use, as these perceptions are closely related to quality of life^(12)^.

## OBJECTIVES

To assess patients’ perceptions regarding PM use, technical concerns, and individual needs after PM implantation.

## METHODS

### Ethical aspects

The research project was developed in accordance with Resolution CNS 466/12 and approved by the Research Ethics Committee of *Escola de Enfermagem de Ribeirão Preto, Universidade de São Paulo* (Certificate of Presentation for Ethical Consideration 45078720.3.0000.5393). Patients were invited to participate. After agreeing, the Informed Consent Form was read and provided in two copies: one for the patient and one archived by the researcher after signing.

### Study design, period, and setting

This is a descriptive cross-sectional observational study. The STrengthening the Reporting of OBservational studies in Epidemiology tool was used to guide the study^(13)^.

### Sample, inclusion and exclusion criteria

The study was conducted in an arrhythmia outpatient clinic of a university hospital in the state of São Paulo. The sample, consecutively and non-probabilistically selected, consisted of patients meeting the following criteria: both sexes; aged over 18 years; regardless of social class or race; and exclusively PM users.

Patients without perception of time, place, and person were excluded. This was assessed using six questions: what is today’s date? What is your age? What day of the week is it? What is the name of this place? What is your full name? What city were you born in?^(14)^. Participants were excluded if they answered three or more questions incorrectly.

### Study protocol

Data collection was carried out on the day of the previously scheduled outpatient follow-up appointment, between September 2023 and May 2024, through individual interviews and review of medical records.

For data collection, a questionnaire was developed containing the following sociodemographic and clinical variables: interview dates, birth dates, and device implantation dates; sex at birth (female or male); presence of a partner (yes or no); education level (in completed years) and professional status (active or inactive); monthly family income (in *reais*); medical diagnosis in the medical record; presence of associated diseases (right heart failure, left heart failure, congestive heart failure, sinus node dysfunction (SND), atrioventricular block (AVB), atrial fibrillation, ischemic cardiomyopathy, Chagas cardiomyopathy, hypertrophic cardiomyopathy, idiopathic dilated cardiomyopathy, arrhythmogenic right ventricular dysplasia, Brugada syndrome, hypertension, diabetes mellitus, dyslipidemia, chronic obstructive pulmonary disease, and coronary artery disease); and current or past smoking (yes or no). The rhythm indicating the device was also collected (SND, first-degree AVB, second-degree AVB, or complete atrioventricular block (CAVB)).

For age calculation, the interview date was subtracted from the date of birth. For PM implant time calculation, the interview date was subtracted from the device implant date.

To assess patients’ perceptions of the artificial cardiac device, a questionnaire developed by Swiss researchers^(15)^ was used in its version adapted for Brazil^(16)^. The questionnaire consists of 23 questions, which assess different aspects of the implanted device (device, techniques, concerns perceptions, and patients’ individual needs). The response options for the 23 questions differ from each other. The results were obtained through simple frequency and percentage of responses to the questions. This data was collected through interviews with participants.

It should be noted that the questionnaire does not measure a subjective construct, but rather assesses everyday situations of people with PM, and there is no score to be obtained.

### Analysis of results and statistics

Data were entered into IBM SPSS version 24.0 for Windows (SPSS, Inc., Chicago, IL, USA) for descriptive analysis of study variables. Simple frequency analyses were performed for nominal or categorical variables, and analyses of central tendency (mean and median) and dispersion (standard deviation) were performed for numerical variables.

## RESULTS

A total of 76 patients participated in the study. To address the objective, a sociodemographic and clinical characterization of participants was performed, followed by a descriptive analysis of patients’ responses to the perception questionnaire. Tables 1 and 2 present the sociodemographic and clinical characteristics.

**Table 1:** Sociodemographic variables of participants (N=76), Ribeirão Preto, São Paulo, Brazil, 2025

Variable	n (%)	Mean (standard deviation)^*^
**Sex**		
Female	40 (52.6)	
Male	36 (47.4)	
**Age**		68.3 (15.4)
**Presence of a companion**		
With companion	43 (56.6)	
Without a companion	33 (43.4)	
**Education level (completed years)**		6.07 (4.4)
**Monthly income (in reais)**		2.260 (1.331)
**Professional situation**		
Inactive	66 (86.8)	
Active	10 (13.2)	

**Table 2:** Clinical variables of participants (N=76), Ribeirão Preto, São Paulo, Brazil, 2025

Heart disease and pacemaker indication rhythm	n (%)
Atrioventricular block	51 (67.1)
Hypertension	29 (38.2)
Chagas cardiomyopathy	21 (27.6)
Sinus node dysfunction	14 (18.4)
Atrial fibrillation	12 (15.8)
Diabetes mellitus	11 (14.5)
Dyslipidemia	8 (10.5)
Congestive heart failure	3 (3.9)
Left heart failure	1 (1.3)
Coronary artery disease	1 (1.3)
**Pacemaker indication rhythm**	
Complete atrioventricular block	43 (56.6)
Sinus node dysfunction	11 (14.5)
Second-degree atrioventricular block	7 (9.2)
First-degree atrioventricular block	1 (1.3)
Not described in the medical record	14 (18.4)

Most participants were female (52.6%), lived with a partner (53.3%), and were not professionally active (86.8%). Regarding the variable “age”, there was a predominance of older adults (over 60 years).

Among the associated cardiac conditions identified in the sample, most patients presented AVB (67.1%), followed by hypertension (38.3%) and Chagas cardiomyopathy (27.6%).

Regarding the indication rhythm for PM implantation, 56.6% of patients presented CAVB, 14.5% SND, 9.2% second-degree AVB, and 1.3% first-degree AVB.

The mean duration since PM implantation was nine years. Among the interviewed patients, 40.8% reported previous smoking, and 6.6% reported continuing smoking even after PM implantation. Tables 3 and 4 present patients’ perceptions regarding PM dependence.

**Table 3:** Responses from participants (N=76) regarding physical perceptions, concerns, and anxiety about pacemaker dependence, Ribeirão Preto, São Paulo, Brazil, 2025

Question	n (%)
**1. To what extent do you feel physically impaired by the implanted device?**	
I do not feel harmed	56 (73.7)
Slightly impaired	12 (15.8)
Severely harmed	8 (10.5)
Totally impaired/incapacitated	Zero
**2. How often do you think about your implanted device?**	
Never	29 (38.2)
Sometimes	13 (17.1)
Several days a week	10 (13.2)
Every day	24 (31.6)
**3. Did you feel depressed when you were informed about the need for the device implant?**	
No	41 (53.9)
Yes, a little	15 (19.7)
Yes, more than a little	10 (13.2)
Yes, a lot	10 (13.2)
**4. Since the device was implanted, to what extent have you been concerned about your heart condition?**	
I am not worried	36 (47.4)
I am a little worried	20 (26.3)
I am more than a little worried	7 (9.2)
I am very worried	13 (17.1)
**5. Did the implanted device change your body image?**	
Yes	21 (27.6)
No	55 (72.4)
**6. To what extent do the visible changes at the implant site bother you?**	
They do not bother me	53 (69.7)
They bother me a little	16 (21.1)
They bother me more than a little	2 (2.6)
They bother me a lot	5 (6.6)
**7. Does the implanted device bother you in your daily life?**	
It does not bother me	55 (72.4)
Yes, it bothers me a little	13 (17.1)
Yes, it bothers me more than a little	6 (7.9)
Yes, it bothers me a lot	2 (2.6)
**8. Does the implanted device bother you during your leisure activities?**	
It does not bother me	56 (73.7)
Yes, it bothers me a little	13 (17.1)
Yes, it bothers me more than a little	3 (3.9)
Yes, it bothers me a lot	4 (5.3)
**9. Do you feel anxious when you think your battery might die sooner than expected?**	
I do not feel anxious	53 (69.7)
Yes, I feel a little	9 (11.8)
Yes, I feel more than a little	2 (2.6)
Yes, I feel very	12 (15.8)
**10. Are you feeling anxious about the possibility of your implanted device malfunctioning?**	
I do not feel anxious	52 (68.4)
Yes, I feel a little	11 (14.5)
Yes, I feel more than a little	3 (3.9)
Yes, I feel very	10 (13.2)

**Table 4:** Responses from participants (N=76) regarding perceptions, information, and psychological support concerning pacemaker dependence, Ribeirão Preto, São Paulo, Brazil, 2025

Question	n (%)
**1. To what extent do you feel informed about the implanted device?**	
Poorly informed	1 (1.3)
Uninformed	13 (17.1)
Very informed	18 (23.7)
Completely informed	44 (57.9)
**2. To what extent do you feel informed about your heart condition?**	
Poorly informed	5 (6.6)
Uninformed	8 (10.5)
Very informed	21 (27.6)
Completely informed	42 (55.3)
**3. To what extent does the implanted device provide you with security?**	
It does not provide security	1 (1.3)
A little	6 (7.9)
More than a little	22 (28.9)
A lot	47 (61.8)
**4. To what extent do you believe the implanted device can prolong your life?**	
I do not consider it	5 (6.6)
A little	6 (7.9)
More than a little	18 (23.7)
A lot	47 (61.8)
**5. Does using an implanted device cause you anxiety?**	
No	65 (85.5)
A little	5 (6.6)
More than a little	4 (5.3)
A lot	2 (2.6)
**6. Would you like to have more frequent appointments with your doctor?**	
Yes	26 (34.2)
No	50 (65.8)
**7. Would you like to have longer appointments with your doctor?**	
Yes	13 (17.1)
No	63 (82.9)
**8. Would you like to have psychological or psychotherapeutic support?**	
Yes	24 (31.6)
No	52 (68.4)
**9. Would you like to join a support group?**	
Yes	28 (36.8)
No	48 (63.2)
**10. Do you believe that people should be better informed about implantable devices for heart disease?**	
Yes	59 (77.6)
No	17 (22.4)
**11. How do you feel now, compared to your state of health before the device was implanted?**	
Worse	4 (5.3)
Equal	21 (27.6)
Better	51 (67.1)
**12. How long did it take you to adapt to the implanted device?**	
Less than one month	45 (59.2)
Up to six months	19 (25.0)
Up to one year	2 (2.6)
Up to two years	5 (6.6)
I have not adapted yet	5 (6.6)
**13. Overall, was it worth having the device implanted?**	
No	3 (3.9)
Probably	2 (2.6)
Yes	71 (93.4)

Positive results were observed regarding physical aspects. Most patients did not feel physically impaired (73.7%) or felt only slightly impaired (15.8%), reporting that they never thought about the PM (38.2%) or only “sometimes” (17.1%). Despite these findings, a portion of patients reported thinking about the device daily (31.6%) or several days per week (13.2%).

Concerning emotional aspects, most patients did not feel depressed when informed about the need for implantation (53.9%) and did not express concern about their cardiac condition (47.4%) or expressed only slight concern (26.3%).

In terms of self-image, most patients (72.4%) reported that the implanted device did not change their body image and were not bothered by visible changes (69.7%).

Regarding daily activities, most participants (72.4%) stated that the PM did not interfere with daily life or interfered only slightly (17.1%). Similarly, it did not interfere with leisure activities (73.7%) or interfered only slightly (17.1%).

Concerning anxiety about complications such as battery lifespan or device malfunction, most patients reported no anxiety (69.7% and 68.4%, respectively). However, most patients reported feeling very secure (61.8%) and believed that the PM would prolong their lives (61.8%).

Most participants reported that psychological/psychotherapeutic support (68.4%) or support groups (63.2%) were not necessary to deal with post-implantation feelings. Most participants also emphasized the importance of information about the cardiac device prior to implantation (77.6%).

The study showed that most patients felt better after PM implantation (67.1%), adapted quickly to the device, and were satisfied with the procedure (93.4%).

## DISCUSSION

This study assessed patients’ perceptions regarding PM use, as well as technical concerns and individual needs after implantation. In the studied sample, most participants were female, lived with a partner, were not professionally active, and were older adults.

The predominance of females corroborates findings from epidemiological studies^(17-19)^. This prevalence may be related to Brazilian demographic characteristics, as women represent more than half of the population according to census data^(20)^. In contrast, studies conducted in Greece^(12)^ and Egypt^(21)^ found a higher prevalence of males, possibly reflecting differences in population structures across countries.

The predominance of individuals over 60 years of age has been reported in studies indicating that advanced age may predispose individuals to CVDs due to fibrosis of the conduction system and a potential future need for artificial electrical stimulation^(8,22)^.

Although CVDs are more frequent among older adults, specialists emphasize that they are not a natural consequence of aging but rather the result of unhealthy habits such as smoking, alcohol consumption, poor sleep, sedentary lifestyle, obesity, and poor control of diabetes and hypertension-major risk factors for CVD development^(2)^. In this study, 40.8% of participants had a history of smoking and 6.6% continued smoking after PM implantation, which may have contributed to the need for electrical stimulation.

The most frequent indication for PM implantation was complete AVB, a conduction disorder in the His bundle. These findings are consistent with another Brazilian study^(22)^ describing the epidemiological profile of patients undergoing PM implantation.

Regarding perceptions of PM dependence, most participants reported not feeling impaired, and the device did not interfere with daily life. However, Brazilian researchers^(23)^ observed worse perceptions regarding the impact of physical condition on work and daily activities.

A study conducted in Turkey^(24)^ found that patients with PM implants (n=50) had lower pulmonary function, respiratory muscle strength, quadriceps and shoulder abductor strength, reduced walking capacity, and greater fatigue and dyspnea compared to healthy controls (n=40). Cardiac rehabilitation programs are recommended for such patients. Even in the absence of perceived physical dependence in this study, healthcare teams should monitor and provide educational interventions when necessary.

Given the inactivity of most participants at the time of data collection, the assessment of the possible interference of PM use in work activities was hampered, as was the real reason for retirement, whether it was due to heart disease or another reason. These factors are considered limitations of this study. Furthermore, authors from Cotonou, Benin^(25)^, found in their study that the main limitations related to the use of PM were difficulty in upper limb mobility related to the device implant, traveling, driving cars, and sexual relations.

Participants felt safe after implantation and recognized the potential severity of their condition without treatment. Most also reported improved well-being after implantation. These findings are consistent with other studies^(25,26)^ demonstrating improved quality of life after PM implantation. These results also support those found in the study by Australian researchers, in which patients showed better quality of life assessments after the device implant^(26)^.

In this study, patients did not feel bothered by the change in body image. This may be related to the long period of use of the device. However, it should be noted that, after the implantation of the artificial cardiac device, people may feel marked both physically and psychologically, requiring them to redefine their routine and reorganize their actions and thoughts^(27)^. Patients reported a different perception of their body image. Reasons included feeling diminished, aesthetic problems related to scarring, and the presence of a foreign body^(25)^. These perceptions should be investigated by healthcare professionals so that, together as a multidisciplinary team, they can provide appropriate support for the reported complaints.

When questioned about their level of information regarding the device and their respective cardiac condition, most participants stated that they were fully informed on these matters. This data is confirmed when questioned about the possibility of more frequent and longer consultations, which was denied in both cases, as participants reported receiving all pertinent information about their condition and the device during the scheduled consultation time.

However, during the interviews, patients reported misconceptions about what they would not be able to do. As a result, they ended up avoiding certain tasks, such as lying on the same side as the PM, moving their arm fully, and using everyday electronic devices. Due to these perceptions observed during the interviews, the need for patient education regarding the PM and their cardiac condition was noted, since a person with a PM can live a life without limitations, provided their clinical condition does not restrict them from certain situations.

This finding is similar to another study in which researchers found that PM implantation improved patients’ quality of life, as they experienced a reduction in symptoms related to discomfort and dyspnea. Therefore, it is of utmost importance to ensure that patients are well-informed about their clinical condition throughout the entire follow-up of their cardiomyopathy^(12)^.

The addition of educational programs can improve the quality of life of patients with PM, providing knowledge and skills to better understand their condition, treatment, and proper management. By learning about lifestyle changes, patients can become more empowered and confident in managing their own health^(28)^. It is essential to carry out educational activities, using illustrated leaflets and personal counseling, to eliminate misconceptions about activities after PM implementation, preventing these myths from resulting in restrictions on daily life^(29)^.

Furthermore, an educational approach targeting the general population would be satisfactory for reducing the stigma of PM dependence and for a better understanding of the feelings that afflict those who use this device. The results showed that, overall, the population has little information about PM.

Another aspect that different professionals should consider when planning the health of patients with chronic diseases is the use of motivational counseling, with the aim of promoting changes in health behavior. A randomized clinical trial, in which patients with permanent PM were randomly allocated to a motivational counseling intervention or to a control group, recorded a statistically significant improvement in mean adherence to care practices, emotional intelligence, and dispositional optimism, as well as a significant reduction in pessimism among the study group compared to the control group^(30)^.

The literature highlights the possibility of developing educational technologies for people with implantable cardiac devices. One example is the podcast “*Alfabeto do Marca-Passo*” (Pacemaker Alphabet), created by Brazilian authors, which presents self-care guidelines from A to Z for the post-implantation period of the device^(31)^. These initiatives are strategies to improve patients’ understanding of health care after implantation.

In this context, the role of nursing stands out as a key player in promoting psychosocial adaptation and coping with the new routines imposed by the use of cardiac PMs. Nurses, as professionals who maintain continuous contact with patients, are in a privileged position to identify doubts, fears, and myths associated with the use of the device, and can intervene in an educational and supportive manner. Active listening and empathetic communication are fundamental resources for strengthening the therapeutic bond and ensuring that guidelines are understood and applied in daily life, promoting autonomy and safety in self-care. In this sense, nursing practice goes beyond the technical dimension, fitting into the broader perspective of care, which recognizes the patient in their physical, emotional, and social entirety.

Additionally, nursing plays a central role in building evidence-based educational strategies, focusing on the prevention of complications and the enhancement of the quality of life of patients with implantable cardiac devices. The development of low-tech care methods, such as discussion groups, educational groups, and audiovisual materials, can be conducted by the nursing team, with a positive impact on treatment adherence and patient empowerment. Recent studies demonstrate that systematized educational interventions, conducted by nurses, have the potential to improve indicators such as self-confidence, therapeutic adherence, and perception of physical and emotional well-being after PM implantation^(30,31)^. Therefore, strengthening the role of nursing in the outpatient and home follow-up of these patients is essential to ensure longitudinal, humanized, and person-centered care.

### Study limitations

This study has limitations that should be considered when interpreting the findings. The sample consisted of patients treated at a single university hospital in the countryside of São Paulo state, selected by convenience and in a non-probabilistic manner, which may compromise the generalization of results and introduce selection bias. The predominance of professionally inactive participants made a more in-depth analysis of the impacts of PM on work activities difficult.

### Contributions to nursing, health, or public policies

As for the contribution that this study makes to nursing science, the nursing team plays a fundamental role in providing post-implantation guidance for PM, especially in guidance on medication management practices, postoperative care with the PM, precautions followed in the postoperative period, dietary modifications, activities of daily living, and follow-up^(21)^.

## CONCLUSIONS

Regarding perceptions and individual needs after implantation, patients assessed PM use positively and reported feeling safe and well informed regarding technical concerns.

## Data Availability

The research data are available within the article.
