# Supplementary material for: Assessment of patients’ perception of the use of an implantable cardiac electronic device
Source: Rev Bras Enferm. 2026 Sep 18;79:e20250299. doi: 10.1590/0034-7167-2025-0299 (PMC13588625; doi:10.1590/0034-7167-2025-0299)
Supplement: Translation

# Translation: Avaliação da percepção dos pacientes sobre o uso de dispositivo eletrônico cardíaco implantável

**DOI:** 10.1590/0034-7167-2025-0299pt

**Authors:** Isabela Santana Oliveira, Renata Elizabete Pagotti da Fonseca, Joyce Aparecida Turco Machado Francisco, Suellen Rodrigues de Oliveira Maier, André Filipe Morais Pinto Novo, Carina Aparecida Marosti Dessotte

**Affiliations:** I Universidade de São Paulo. Ribeirão Preto, São Paulo, Brasil; II Universidade Federal de São Carlos. São Carlos, São Paulo, Brasil; III Universidade Federal de Rondonópolis. Rondonópolis, Mato Grosso, Brasil; IV Instituto Politécnico de Bragança. Bragança, Portugal

**Keywords:** Marca-Passo Artificial, Percepção, Enfermagem Perioperatória, Arritmias Cardíacas, Doenças Cardiovasculares.

## Abstract

**Objetivos:**

avaliar as percepções dos pacientes após o implante do marca-passo.

**Métodos:**

estudo observacional descritivo, transversal, realizado em hospital universitário do interior paulista, com uma amostra consecutiva e não probabilística de pacientes adultos portadores de marca-passo. Utilizou-se um questionário de avaliação das percepções dos pacientes sobre o marca-passo, com análise de dados obtida por frequência simples e porcentagem de respostas.

**Resultados:**

dos 76 pacientes entrevistados, a maioria era do sexo feminino, tinha companheiro, era inativa, tinha baixa escolaridade e renda familiar, com idade média de 68,3 anos. O principal motivo para a indicação do implante foi o bloqueio atrioventricular total. A maioria (73,7%) não apresentou sentimento de prejuízo físico, nem se sentiu deprimida ou ansiosa. Também não relataram alteração na imagem corporal, nas atividades diárias ou de lazer. Além disso, demonstraram muita segurança quanto ao uso do dispositivo (61,8%).

**Conclusões:**

os pacientes avaliaram de forma positiva o uso do marca-passo.

## INTRODUÇÃO

As doenças cardiovasculares (DCVs) são a principal causa de mortes no mundo. Entre 1990 e 2022, os óbitos por DCVs aumentaram de 12,4 milhões para 19,8 milhões, enquanto a população mundial cresceu 51%^(1)^. No Brasil, as DCVs também lideram entre as causas. Após perderem a posição para a COVID-19 em 2021, as doenças do coração e do sistema circulatório voltaram ao topo, com cerca de 400 mil brasileiros falecendo em 2022 devido a 18 tipos de DCVs, segundo o relatório “Carga global de doenças e fatores de risco cardiovasculares”^(2)^.

Atualmente, no Brasil, estima-se que mais de 20 milhões de pessoas tenham algum tipo de arritmia cardíaca^(3)^. Segundo os dados do Registro Brasileiro de Marcapassos, Ressincronizadores e Desfibriladores, no período de 12 de julho de 2024 a 12 de agosto de 2024, foram realizados 1.723 implantes de marca-passo (MCP) em território nacional^(4)^. De janeiro a junho de 2024, foram realizados no total 435.211 implantes de MCP, sendo a região Sudeste a que mais realizou implantes (196.377), seguida pelas regiões Sul (101.713), Nordeste (82.035), Centro-Oeste (40.203) e Norte (14.883)^(5)^.

O paciente com indicação de MCP apresenta DCV grave e incompatível com a vida, sendo o implante muitas vezes uma das últimas opções terapêuticas. Após o implante, podem surgir complicações tanto da deterioração cardíaca quanto do próprio dispositivo. Além das infecções, há riscos de cardiomiopatia induzida, regurgitação tricúspide, perfuração cardíaca, hemotórax e pneumotórax^(6)^. Esses problemas podem ser minimizados com a adesão a cuidados específicos para preservar o dispositivo^(7)^.

Estimativas globais indicam que 1,25 milhões de MCPs permanentes são implantados anualmente, e espera-se que esse número aumente nos próximos anos^(8)^. Diante dessa tendência global, deve-se prestar atenção especial ao impacto da terapia com uso do MCP na qualidade de vida desses pacientes e na percepção que eles possuem sobre essa terapêutica.

Pacientes com MCPs mais antigos podem enfrentar interferências ambientais que, embora raras, podem afetar seu funcionamento. Essas interferências podem causar inibição do estímulo, deflagração inadequada, funcionamento assíncrono, mudança de programação e aceleração indevida. Dependendo da intensidade, podem resultar em disfunção transitória, dano irreversível ao gerador, arritmias e alterações na junção eletrodo-coração^(9)^.

Embora essas interferências possam ocorrer, o MCP em si não traz restrições de atividade física, permitindo o retorno do paciente à sua vida cotidiana. A falta de conhecimento ou a compreensão incorreta podem resultar em restrições desnecessárias ou riscos secundários à interferência, prejudicando a qualidade de vida dos pacientes^(10)^.

Em uma perspectiva social, manter a qualidade de vida cria estabilidade e igualdade entre os membros da sociedade como um todo. Com relação ao paciente, a qualidade de vida reflete o que realmente acontece em suas vidas diárias e indica a diferença entre suas esperanças e expectativas. E em uma perspectiva clínica, a qualidade de vida fornece informações essenciais para os profissionais de saúde ao planejar práticas de cuidado centradas no paciente^(11)^. Assim, torna-se salutar um olhar para as percepções dos pacientes com relação ao uso do MCP, já que essas percepções têm estreita relação com a qualidade de vida^(12)^.

## OBJETIVOS

Avaliar as percepções dos pacientes sobre o MCP, as preocupações técnicas e as necessidades individuais após o implante do MCP.

## MÉTODOS

### Aspectos éticos

O projeto de pesquisa foi elaborado de acordo com os preceitos da Resolução CNS n^o^ 466/12, e foi aprovado pelo Comitê de Ética em Pesquisa da Escola de Enfermagem de Ribeirão Preto da Universidade de São Paulo (Certificado de Apresentação para Apreciação Ética n^o^ 45078720.3.0000.5393). Os pacientes foram convidados a participar da pesquisa. Após concordarem, foi lido o Termo de Consentimento Livre e Esclarecido, que foi fornecido em duas vias: uma para o paciente e a outra foi arquivada pelo pesquisador, após as assinaturas.

### Desenho, período e local do estudo

Trata-se de estudo observacional descritivo de corte transversal. Para orientar a condução do estudo, foi utilizada a ferramenta *STrengthening the Reporting of OBservational studies in Epidemiology* ^(13)^.

### Amostra, critérios de inclusão e exclusão

O estudo foi realizado em um ambulatório de arritmia de hospital universitário no interior paulista. A amostra, constituída de forma consecutiva e não probabilística, foi composta por pacientes que atenderam aos seguintes critérios de inclusão: ambos os sexos; com idade acima de 18 anos; independentemente da classe social e da raça; e portadores de MCP exclusivamente.

Foram excluídos os pacientes que não tiveram a percepção de tempo, espaço e pessoa. Para essa avaliação, foram utilizadas seis questões: qual a data de hoje? Qual a sua idade? Em que dia da semana estamos? Qual o nome do local que estamos nesse momento? Qual o seu nome completo? Qual o nome da cidade em que você nasceu?^(14)^. Os participantes foram excluídos do estudo quando erraram ou não souberam responder três ou mais questões.

### Protocolo do estudo

A coleta de dados foi realizada no dia do retorno ambulatorial, previamente agendado, entre os meses de setembro de 2023 e maio de 2024, por meio de entrevistas individuais e consulta aos prontuários.

Para a coleta de dados, foi elaborado um questionário contendo as seguintes variáveis sociodemográficas e clínicas: datas da entrevista, de nascimento e de implante do dispositivo; sexo ao nascer (feminino ou masculino); presença de companheiro (sim ou não); escolaridade (em anos completos) e situação profissional (ativo ou inativo); renda mensal familiar (em reais); diagnóstico médico no prontuário; presença de doenças associadas (insuficiência cardíaca direita, insuficiência cardíaca esquerda, insuficiência cardíaca congestiva, disfunção do nó sinusal (DNS), bloqueio atrioventricular (BAV), fibrilação atrial, cardiomiopatia isquêmica, cardiomiopatia chagásica, cardiomiopatia hipertrófica, cardiomiopatia dilatada idiopática, displasia arritmogênica do ventrículo direito, síndrome de Brugada, hipertensão arterial sistêmica, diabetes mellitus, dislipidemia, doença pulmonar obstrutiva crônica e doença arterial coronariana); e tabagismo atual ou pregresso (sim ou não). Foi coletado também o ritmo de indicação do dispositivo (DNS, BAV de 1º grau, BAV de 2º grau ou bloqueio atrioventricular total (BAVT)).

Para o cálculo da idade, a data da entrevista foi subtraída da data de nascimento. Para o cálculo do tempo de implante do MCP, a data da entrevista foi subtraída da data do implante do dispositivo.

Para a avaliação das percepções dos pacientes sobre o dispositivo cardíaco artificial, foi utilizado questionário elaborado por pesquisadores suíços^(15)^ na sua versão adaptada para o Brasil^(16)^. O questionário é composto por 23 perguntas, que avaliam diferentes aspectos do dispositivo implantado (percepções do dispositivo, técnicas, preocupações e necessidades individuais dos pacientes). As opções de resposta das 23 perguntas diferem entre si. Os resultados foram obtidos por meio de frequência simples e porcentagem de respostas às questões. Esses dados foram coletados por meio de entrevista com os participantes.

Ressalta-se que o questionário não mede um construto subjetivo, mas avalia situações cotidianas de pessoas com MCP, e não há um escore a ser obtido.

### Análise dos resultados e estatística

Os dados foram inseridos no programa IBM SPSS, versão 24.0, para *Windows* (SPSS, Inc., Chicago, IL, USA), para a realização da análise descritiva das variáveis do estudo. Análises de frequência simples foram realizadas para variáveis nominais ou categóricas, e análises de tendência central (média e mediana) e de dispersão (desvio padrão), para variáveis numéricas.

## RESULTADOS

Participaram do estudo 76 pacientes. Para responder ao objetivo, foi realizada a caracterização sociodemográfica e clínica dos participantes, seguida pela análise descritiva das respostas dos pacientes ao questionário sobre as percepções. As Tabelas 1 e 2 mostram a caracterização sociodemográfica e clínica.

**Tabela 1:** Variáveis sociodemográficas dos participantes (N=76), Ribeirão Preto, São Paulo, Brasil, 2025

Variável	n (%)	Média (desvio padrão)^*^
**Sexo**		
Feminino	40 (52,6)	
Masculino	36 (47,4)	
**Idade**		68,3 (15,4)
**Presença de companheiro**		
Com companheiro	43 (56,6)	
Sem companheiro	33 (43,4)	
**Escolaridade (anos completos)**		6,07 (4,4)
**Renda mensal (em reais)**		2.260 (1.331)
**Situação profissional**		
Inativo	66 (86,8)	
Ativo	10 (13,2)	

**Tabela 2:** Variáveis clínicas dos participantes (N=76), Ribeirão Preto, São Paulo, Brasil, 2025

Doenças cardíacas e ritmo de indicação do marca-passo	n (%)
Bloqueio atrioventricular	51 (67,1)
Hipertensão arterial sistêmica	29 (38,2)
Cardiomiopatia chagásica	21 (27,6)
Disfunção do nó sinusal	14 (18,4)
Fibrilação atrial	12 (15,8)
Diabetes mellitus	11 (14,5)
Dislipidemia	8 (10,5)
Insuficiência cardíaca congestiva	3 (3,9)
Insuficiência cardíaca à esquerda	1 (1,3)
Doença arterial coronariana	1 (1,3)
**Ritmo de indicação do marca-passo**	
Bloqueio atrioventricular total	43 (56,6)
Disfunção do nó sinusal	11 (14,5)
Bloqueio atrioventricular de 2° grau	7 (9,2)
Bloqueio atrioventricular de 1° grau	1 (1,3)
Não descrito no prontuário	14 (18,4)

A maioria dos participantes entrevistados era do sexo feminino (52,6%), vivia com companheiro (53,3%) e era inativa (86,8%). Quanto à variável “idade”, houve predominância de pacientes idosos (acima de 60 anos).

Quanto às doenças cardíacas associadas encontradas na amostra estudada, a maioria dos pacientes apresentou BAV (67,1%), seguida por hipertensão arterial sistêmica (38,3%) e cardiomiopatia chagásica (27,6%).

Com relação ao ritmo de indicação do implante do MCP, foi encontrado que 56,6% dos pacientes apresentaram BAVT, 14,5%, DNS, 9,2%, BAV de 2º grau, e 1,3%, BAV de 1º grau.

O tempo médio de implante do MCP foi de nove anos. Dos pacientes entrevistados, 40,8% referiram tabagismo pregresso, e 6,6% relataram continuar com o tabagismo, mesmo após o implante de MCP. Nas Tabelas 3 e 4, encontram-se as percepções dos pacientes acerca da dependência do MCP.

**Tabela 3:** Respostas dos participantes (N=76) sobre as percepções físicas, preocupações e ansiedade quanto à dependência do marca-passo, Ribeirão Preto, São Paulo, Brasil, 2025

Questões	n (%)
**1. Até que ponto você se sente fisicamente prejudicado pelo aparelho implantado?**	
Não me sinto prejudicado	56 (73,7)
Um pouco prejudicado	12 (15,8)
Muito prejudicado	8 (10,5)
Totalmente prejudicado/incapacitado	Zero
**2. Com que frequência você pensa no seu aparelho implantado?**	
Nunca	29 (38,2)
Às vezes	13 (17,1)
Vários dias por semana	10 (13,2)
Todos os dias	24 (31,6)
**3. Você se sentiu deprimido quando foi informado sobre a necessidade do implante do aparelho?**	
Não	41 (53,9)
Sim, um pouco	15 (19,7)
Sim, mais do que pouco	10 (13,2)
Sim, muito	10 (13,2)
**4. Desde o implante do aparelho, até que ponto você se preocupa com sua condição cardíaca?**	
Não me preocupo	36 (47,4)
Preocupo-me um pouco	20 (26,3)
Preocupo-me mais do que um pouco	7 (9,2)
Preocupo-me muito	13 (17,1)
**5. O aparelho implantado mudou a imagem que você tinha do seu corpo?**	
Sim	21 (27,6)
Não	55 (72,4)
**6. Até que ponto as mudanças visíveis no local do implante do aparelho incomodam você?**	
Não me incomodam	53 (69,7)
Incomodam-me um pouco	16 (21,1)
Incomodam-me mais do que um pouco	2 (2,6)
Incomodam-me muito	5 (6,6)
**7. O aparelho implantado incomoda você em sua vida diária?**	
Não me incomoda	55 (72,4)
Sim, incomoda-me um pouco	13 (17,1)
Sim, incomoda-me mais do que um pouco	6 (7,9)
Sim, incomoda-me muito	2 (2,6)
**8. O aparelho implantado incomoda você em suas atividades de lazer?**	
Não me incomoda	56 (73,7)
Sim, incomoda-me um pouco	13 (17,1)
Sim, incomoda-me mais do que um pouco	3 (3,9)
Sim, incomoda-me muito	4 (5,3)
**9. Você se sente ansioso ao pensar que a bateria pode acabar antes do previsto?**	
Não me sinto ansioso	53 (69,7)
Sim, sinto-me um pouco	9 (11,8)
Sim, sinto-me mais que um pouco	2 (2,6)
Sim, sinto-me muito	12 (15,8)
**10. Você se sente ansioso em relação ao mau funcionamento do aparelho implantado?**	
Não me sinto ansioso	52 (68,4)
Sim, sinto-me um pouco	11 (14,5)
Sim, sinto-me mais que um pouco	3 (3,9)
Sim, sinto-me muito	10 (13,2)

**Tabela 4:** Respostas dos participantes (N=76) sobre as percepções, informações e apoio psicológico quanto à dependência do marca-passo, Ribeirão Preto, São Paulo, Brasil, 2025

Questão	n (%)
**1. Até que ponto você se sente informado sobre o aparelho implantado?**	
Mal-informado	1 (1,3)
Pouco informado	13 (17,1)
Muito informado	18 (23,7)
Totalmente informado	44 (57,9)
**2. Até que ponto você se sente informado sobre a sua condição cardíaca?**	
Mal-informado	5 (6,6)
Pouco informado	8 (10,5)
Muito informado	21 (27,6)
Totalmente informado	42 (55,3)
**3. Até que ponto o aparelho implantado traz segurança para você?**	
Não traz segurança	1 (1,3)
Um pouco	6 (7,9)
Mais que um pouco	22 (28,9)
Muita	47 (61,8)
**4. Até que ponto você considera que o aparelho implantado pode prolongar sua vida?**	
Não considero	5 (6,6)
Um pouco	6 (7,9)
Mais que um pouco	18 (23,7)
Muito	47 (61,8)
**5. Usar um aparelho implantado causa ansiedade em você?**	
Não	65 (85,5)
Um pouco	5 (6,6)
Mais que um pouco	4 (5,3)
Muita	2 (2,6)
**6. Você gostaria de ter consultas mais frequentes com seu médico?**	
Sim	26 (34,2)
Não	50 (65,8)
**7. Você gostaria de ter consultas mais longas com seu médico?**	
Sim	13 (17,1)
Não	63 (82,9)
**8. Você gostaria de ter apoio psicológico ou psicoterapêutico?**	
Sim	24 (31,6)
Não	52 (68,4)
**9. Você gostaria de participar de um grupo de apoio?**	
Sim	28 (36,8)
Não	48 (63,2)
**10. Você acredita que as pessoas deveriam ser mais bem informadas sobre aparelhos implantáveis para doenças cardíacas?**	
Sim	59 (77,6)
Não	17 (22,4)
**11. Como você se sente agora, em comparação com seu estado de saúde antes da implantação do aparelho?**	
Pior	4 (5,3)
Igual	21 (27,6)
Melhor	51 (67,1)
**12. Quanto tempo você levou para se adaptar ao aparelho implantado?**	
Menos de um mês	45 (59,2)
Até seis meses	19 (25,0)
Até um ano	2 (2,6)
Até dois anos	5 (6,6)
Ainda não me adaptei	5 (6,6)
**13. De forma geral, valeu a pena ter implantado o aparelho?**	
Não	3 (3,9)
Provavelmente	2 (2,6)
Sim	71 (93,4)

Encontraram-se resultados positivos com relação aos aspectos físicos. A maioria não se sentiu prejudicada fisicamente (73,7%) ou se sentiu pouco prejudicada (15,8%), relatando que nunca parou para pensar no MCP (38,2%) ou só “às vezes” (17,1%). Apesar desses resultados, uma parcela dos pacientes entrevistados relatou que ainda pensa todos os dias (31,6%) ou vários dias por semana (13,2%) no dispositivo.

Acerca dos aspectos emocionais, a maioria dos pacientes não se sentiu deprimida quando foi informada sobre a necessidade do implante (53,9%), não demonstrou preocupação com sua condição cardíaca (47,4%) ou demonstrou pouca preocupação (26,3%).

Em relação aos aspectos de autoimagem, a maioria (72,4%) informou que o aparelho implantado não mudou a autoimagem e não se incomodou com as mudanças visíveis (69,7%).

No tocante às atividades realizadas, a maioria (72,4%) declarou que o MCP não incomodou na sua vida diária ou incomodou pouco (17,1%). Também avaliou que não incomodou nas atividades de lazer (73,7%) ou incomodou pouco (17,1%).

Sobre o sentimento de ansiedade ao pensar em complicações como o tempo de vida útil do gerador do MCP ou mau funcionamento do dispositivo, a maioria alegou não se incomodar (69,7% e 68,4%, respectivamente). Todavia, a maioria dos pacientes explanou sentir muita segurança (61,8%) e considerou que o MCP irá prolongar sua vida (61,8%).

A maioria avaliou que não seria necessário apoio psicológico/psicoterapêutico (68,4%) ou grupos de apoio (63,2%) para lidar com os sentimentos após a implantação do MCP. A maioria dos entrevistados acreditava na importância da informação sobre o dispositivo cardíaco previamente ao implante (77,6%).

O estudo evidenciou que a maioria dos pacientes se sentiu melhor com a implantação do MCP (67,1%), adaptou-se rapidamente ao aparelho implantado e estava satisfeita com a realização do procedimento (93,4%).

## DISCUSSÃO

No presente estudo, foram avaliadas as percepções dos pacientes sobre o uso do MCP, bem como as preocupações técnicas e as necessidades individuais após o implante. Na amostra estudada, observou-se que a maioria dos participantes era do sexo feminino, vivia com companheiro, estava inativa no momento da coleta de dados e era idosa.

A predominância do sexo feminino corrobora os resultados encontrados em estudos epidemiológicos^(17-19)^. Esta prevalência pode se relacionar à questão demográfica brasileira, tendo em vista que, no Censo Demográfico, mais da metade da população brasileira é composta por mulheres^(20)^. Contrastando com esse resultado, estudos realizados na Grécia^(12)^ e no Egito^(21)^ evidenciaram maior prevalência do sexo masculino, o que pode ter relações com as características censitárias conforme as características dos diferentes países.

A prevalência de participantes acima de 60 anos foi citada em estudos que afirmaram que uma idade mais avançada pode ser um fator predisposto para DCVs, devido a uma fibrose do sistema de condução e uma possível necessidade futura de estimulação elétrica artificial^(8,22)^.

Apesar de a frequência de DCVs ser maior na população idosa, especialistas afirmam que elas não são uma característica natural do envelhecimento, mas uma consequência de danos provocados por hábitos pouco saudáveis (fumar, consumir bebidas alcoólicas, dormir mal, ser sedentário, excesso de peso, pouco controle do diabetes e da hipertensão arterial), sendo os principais fatores de risco para o desenvolvimento das DCVs^(2)^. Entre os participantes do estudo, 40,8% tinham histórico de tabagismo e 6,6% mantiveram o hábito mesmo após o implante de MCP. Esse achado pode ter favorecido a necessidade de estimulação elétrica.

O motivo mais frequente de implante de MCP observado entre os participantes foi BAVT. Trata-se de um problema na condução do estímulo elétrico no Feixe de His. Esses dados corroboram os resultados de outro estudo brasileiro^(22)^, no qual foi apresentado o perfil epidemiológico de pacientes submetidos à implantação de MCP.

Com relação às percepções sobre a dependência do MCP, os participantes predominantemente afirmaram não se sentirem prejudicados, e a maioria afirmou que o aparelho implantado não incomodava na vida diária. Entretanto, pesquisadores brasileiros^(23)^ observaram que os pacientes tiveram uma avaliação pior em relação ao impacto de sua condição física nas atividades laborais e diárias.

Em estudo realizado na Turquia^(24)^, os autores observaram que pacientes com implante de MCP (n=50) apresentaram menor função pulmonar, força muscular respiratória, de quadríceps e de abdutores do ombro, além de capacidade de caminhada reduzida e maior percepção de fadiga e dispneia, em comparação ao grupo controle saudável (n=40). Recomenda-se a inclusão desses pacientes em programas de reabilitação cardíaca. Mesmo sem a percepção de dependência física neste estudo, a equipe de saúde deve monitorar e intervir, educativamente, quando necessário.

Diante da inatividade da maioria dos participantes no momento da coleta de dados, a avaliação acerca da possível interferência do uso do MCP nas atividades laborais foi prejudicada, bem como o real motivo da aposentadoria, se foi decorrente da doença cardíaca ou de outro motivo. Tais fatores são considerados limitações deste estudo. Ademais, autores de Cotonou, no Bénin^(25)^, encontraram, em seu estudo, que as principais limitações relacionadas ao uso do MCP foram a dificuldade de mobilidade do membro superior referente ao implante do dispositivo, a realização de viagens, a condução de automóveis e as relações sexuais. Inclusive, nesse estudo, um paciente relatou que seu divórcio foi, inteiramente, devido ao medo de danificar o MCP.

Os participantes do estudo se sentiram seguros com o implante e identificaram a possibilidade de uma situação clínica mais grave na ausência desse tratamento. A maioria também se sentiu melhor após o implante do MCP. Esses dados corroboram os de outro estudo^(25)^, no qual a maioria dos pacientes tinha boa percepção geral do seu estado de saúde após o implante do MCP. Esses resultados corroboram igualmente com os encontrados no estudo de pesquisadores australianos, no qual os pacientes apresentaram melhorias significativas na qualidade de vida após o implante do dispositivo^(26)^.

Neste estudo, os pacientes não se sentiram incomodados com a mudança da imagem corporal. Esse ponto pode estar relacionado ao longo tempo de uso do aparelho. Contudo, deve-se observar que, após o implante do dispositivo cardíaco artificial, a pessoa pode se sentir marcada tanto física quanto psicologicamente, sendo necessário ressignificar sua rotina e reorganizar suas ações e pensamentos^(27)^. Pacientes relataram uma percepção diferente da imagem corporal. As razões incluíam se sentir diminuído, problemas estéticos relacionados à cicatriz e a presença de um corpo estranho^(25)^. Essas percepções devem ser investigadas pelos profissionais de saúde, para que, juntos, como equipe multiprofissional, possam dar o suporte adequado às queixas relatadas.

Quando questionados sobre o grau de informação que possuíam acerca do dispositivo e da sua respectiva condição cardíaca, afirmavam estar, em sua maioria, totalmente informados sobre esses quesitos. Esses dados são confirmados ao serem questionados acerca da possibilidade de consultas mais frequentes e mais longas, o que foi negado em ambos os casos, visto que os participantes relataram receber todas as informações pertinentes ao seu quadro e sobre o dispositivo durante o tempo previsto da consulta.

Entretanto, durante as entrevistas, os pacientes relataram percepções errôneas sobre o que não seria possível realizar. Desse modo, acabavam por evitar algumas tarefas, como deitar-se do mesmo lado do MCP, mover o braço totalmente e utilizar aparelhos eletrônicos do uso cotidiano. Devido a essas percepções, observadas durante as entrevistas, notou-se a necessidade de orientação do paciente quanto ao MCP e à sua condição cardíaca, uma vez que o portador de MCP pode ter uma vida sem limitações, desde que sua condição clínica não seja restritiva de situações limitantes.

Esse achado se assemelha a de outro estudo, no qual os pesquisadores encontraram que o implante de MCP melhorou a qualidade de vida dos pacientes, uma vez que estes perceberam a diminuição dos sintomas relacionados ao desconforto e à dispneia. Diante do exposto, é de suma importância garantir que os pacientes estejam bem-informados sobre a sua condição clínica durante todo acompanhamento da cardiomiopatia^(12)^.

A adição de programas educativos pode melhorar a qualidade de vida dos pacientes com MCP, proporcionando conhecimentos e competências para melhor compreender a sua condição, tratamento e gestão adequada. Ao aprender sobre as mudanças no estilo de vida, os pacientes podem se tornar mais capacitados e confiantes na gestão da sua própria saúde^(28)^. É fundamental realizar atividades educativas, utilizando folhetos ilustrados e aconselhamento pessoal, para eliminar conceitos errôneos sobre atividades após a implantação de MCP, evitando que esses mitos resultem em restrições à vida cotidiana^(29)^.

Além disso, uma abordagem educativa da população geral seria satisfatória para a diminuição do estigma de depender do MCP e para uma melhor compreensão dos sentimentos que afligem os portadores desse dispositivo. Os resultados mostraram que, no geral, a população possui pouca informação sobre o MCP.

Outro aspecto que os diferentes profissionais devem considerar no planejamento da saúde de pacientes com doenças crônicas é o uso de aconselhamento motivacional, com o objetivo de promover mudanças no comportamento de saúde. Ensaio clínico randomizado, no qual pacientes com MCP permanentes foram aleatoriamente alocados para uma intervenção de aconselhamento motivacional ou para um grupo controle, registrou uma melhoria estatisticamente significativa nas médias de adesão às práticas de cuidados, de inteligência emocional e de otimismo disposicional, bem como uma redução significativa no pessimismo entre o grupo de estudo, em comparação ao grupo de controle^(30)^.

A literatura evidencia a possibilidade de desenvolvimento de tecnologias educacionais para pessoas com dispositivos cardíacos implantáveis. Um exemplo é o *podcast* “Alfabeto do Marca-Passo”, criado por autores brasileiros, que apresenta diretrizes de autocuidado de A a Z para o período de pós-implantação do dispositivo^(31)^. Essas iniciativas são estratégias para melhorar a compreensão dos pacientes sobre cuidados de saúde após a implantação.

Nesse contexto, destaca-se o papel da enfermagem como protagonista na promoção da adaptação psicossocial e no enfrentamento das novas rotinas impostas pelo uso do MCP cardíaco. O enfermeiro, como profissional que mantém contato contínuo com o paciente, está em posição privilegiada para identificar dúvidas, medos e mitos associados ao uso do dispositivo, podendo intervir de forma educativa e acolhedora. A escuta qualificada e a comunicação empática são recursos fundamentais para fortalecer o vínculo terapêutico e garantir que as orientações sejam compreendidas e aplicadas no cotidiano, promovendo autonomia e segurança no autocuidado. A atuação da enfermagem, nesse sentido, vai além da dimensão técnica, inserindo-se na perspectiva ampliada do cuidado, que reconhece o paciente em sua integralidade física, emocional e social.

Adicionalmente, a enfermagem tem papel central na construção de estratégias educativas baseadas em evidências, com foco na prevenção de complicações e na valorização da qualidade de vida dos pacientes com dispositivos cardíacos implantáveis. O desenvolvimento de tecnologias leves de cuidado, como rodas de conversa, grupos educativos e materiais audiovisuais, pode ser conduzido pela equipe de enfermagem, com impacto positivo sobre a adesão ao tratamento e o empoderamento do paciente. Estudos recentes demonstram que intervenções educativas sistematizadas, conduzidas por enfermeiros, têm potencial para melhorar indicadores como autoconfiança, adesão terapêutica e percepção de bem-estar físico e emocional após o implante de MCP^(30,31)^. Dessa forma, o fortalecimento do papel da enfermagem no seguimento ambulatorial e domiciliar desses pacientes é essencial para garantir um cuidado longitudinal, humanizado e centrado na pessoa.

### Limitações do estudo

Este estudo apresenta limitações que devem ser consideradas na interpretação dos achados. A amostra foi composta por pacientes atendidos em um único hospital universitário do interior paulista, selecionados por conveniência e de forma não probabilística, o que pode comprometer a generalização dos resultados e introduzir viés de seleção. A predominância de participantes inativos profissionalmente dificultou a análise mais aprofundada sobre os impactos do MCP nas atividades laborais.

### Contribuições para a área da enfermagem, saúde ou políticas públicas

Com relação à contribuição que este estudo traz à ciência da enfermagem, a equipe de enfermagem desempenha papel fundamental na realização de orientações pós-implante de MCP, em especial na orientação sobre as práticas de gerenciamento de medicação, cuidados pós-operatórios com o MCP, precauções seguidas no pós-operatório, modificações da dieta, atividades da vida diária e acompanhamento^(21)^.

## CONCLUSÕES

Quanto às percepções e às necessidades individuais após o implante, os pacientes avaliaram de forma positiva o uso do MCP, e estavam seguros e bem-informados com relação às preocupações técnicas.

## Data Availability

Os dados de pesquisa estão disponíveis no corpo do artigo.
